# A 61-Year-Old Male With Chronic Appendicitis: A Case Report

**DOI:** 10.7759/cureus.32130

**Published:** 2022-12-02

**Authors:** Omar Almansouri, Abdulrahman M Algethmi, Majed Qutub, Muhammad A Khan, Nadia Mazraani

**Affiliations:** 1 Medicine and Surgery, King Saud Bin Abdulaziz University for Health Sciences, Jeddah, SAU; 2 Medical Student, King Saud Bin Abdulaziz University for Health Sciences College of Medicine, Jeddah, SAU; 3 Medicine and Surgery, King Saud Bin Abdulaziz University for Health Sciences College of Medicine, Jeddah, SAU; 4 Medical Education, King Saud Bin Abdulaziz University for Health Sciences, Jeddah, SAU; 5 Family and Community Medicine, King Abdulaziz Medical City Riyadh, Jeddah, SAU; 6 Family and Community Medicine, King Saud Bin Abdulaziz University for Health Sciences College of Medicine, Jeddah, SAU

**Keywords:** chronic abdominal pain, atypical appendicitis, vague abdominal pain, periumbilical pain, chronic appendicitis

## Abstract

The objective of this report is to present a rare case of chronic appendicitis with an atypical presentation. The patient presented with intermittent periumbilical pain without any other symptom, relieved by an anti-inflammatory, and later incidentally diagnosed on computed tomography (CT) scan. The patient was managed with an appendectomy during laparoscopic exploration.

A 61-year-old male, with a history of renal calculi, managed type 2 diabetes mellitus, managed hypertension, and ischemic heart disease who underwent percutaneous coronary intervention 10 years ago, presented to the clinic for a CT scan to follow up a non-obstructing renal calculus diagnosed previously. The imaging showed incidental appendiceal findings, and the patient informed the medical team that he had been experiencing intermittent periumbilical pain once every 4-12 weeks for the past year, which was not associated with fever, nausea, or vomiting. At that time, oral non-steroidal anti-inflammatory drugs (NSAIDs) were prescribed, following which his symptoms subsided. A few days later, he presented to the clinic with the same complaint. The patient underwent laparoscopic exploration after numerous clinic visits and was diagnosed with chronic appendicitis.

Chronic appendicitis should be explored in afebrile patients with periumbilical pain lasting for several days without other symptoms or predisposing factors. It should also be suspected in patients with recurrent or intermittent vague abdominal pain that subsides with NSAIDs. A CT scan of the abdomen should be conducted, and if the results confirm or imply chronic appendicitis, appendectomy is the preferred therapy.

## Introduction

One of the most common abdominal surgical emergencies is appendicitis, which affects approximately 10% of the general population and most often presents at the ages of 10-30 years [1]. The classical symptoms of acute appendicitis present within 48 hours as periumbilical pain localizing to the right iliac fossa near McBurney’s point. The pain is associated with anorexia, guarding, and high white cell count [2]. However, a far less common presentation occurs as chronic appendicitis, accounting for 1-1.5% of all cases of appendicitis [2-4]. Although the exact pathophysiology of chronic appendicitis is less understood, traditionally it has been defined as a long-standing inflammation or fibrosis displayed as right lower quadrant (RLQ) pain for more than 48 hours, or intermittent in nature. Patients with chronic appendicitis may present with recurring RLQ abdominal pain unrelated to any febrile infection, or with self-limiting pain that resolves on its own [5]. These instances are usually less severe than acute appendicitis, and their symptoms are frequently non-specific or unusual for appendicitis [5]. Chronic appendicitis is treated with appendectomy and is not considered a surgical emergency [2,6]. It can, however, remain untreated or misdiagnosed, leading to consequences such as perforation, abscess formation, peritonitis, and infertility. The majority of these complications necessitate surgical intervention [1,2,7].

Therefore, we present this case of a 61-year-old male who presented with a one-year history of intermittent periumbilical pain, not associated with fever, nausea, or vomiting. During the one-year period, the patient was investigated with various imaging techniques that showed different results. He later presented with abdominal pain and positive ultrasound (US) findings for an enlarged appendix, which was surgically treated as chronic appendicitis.

## Case presentation

**Figure 1:**
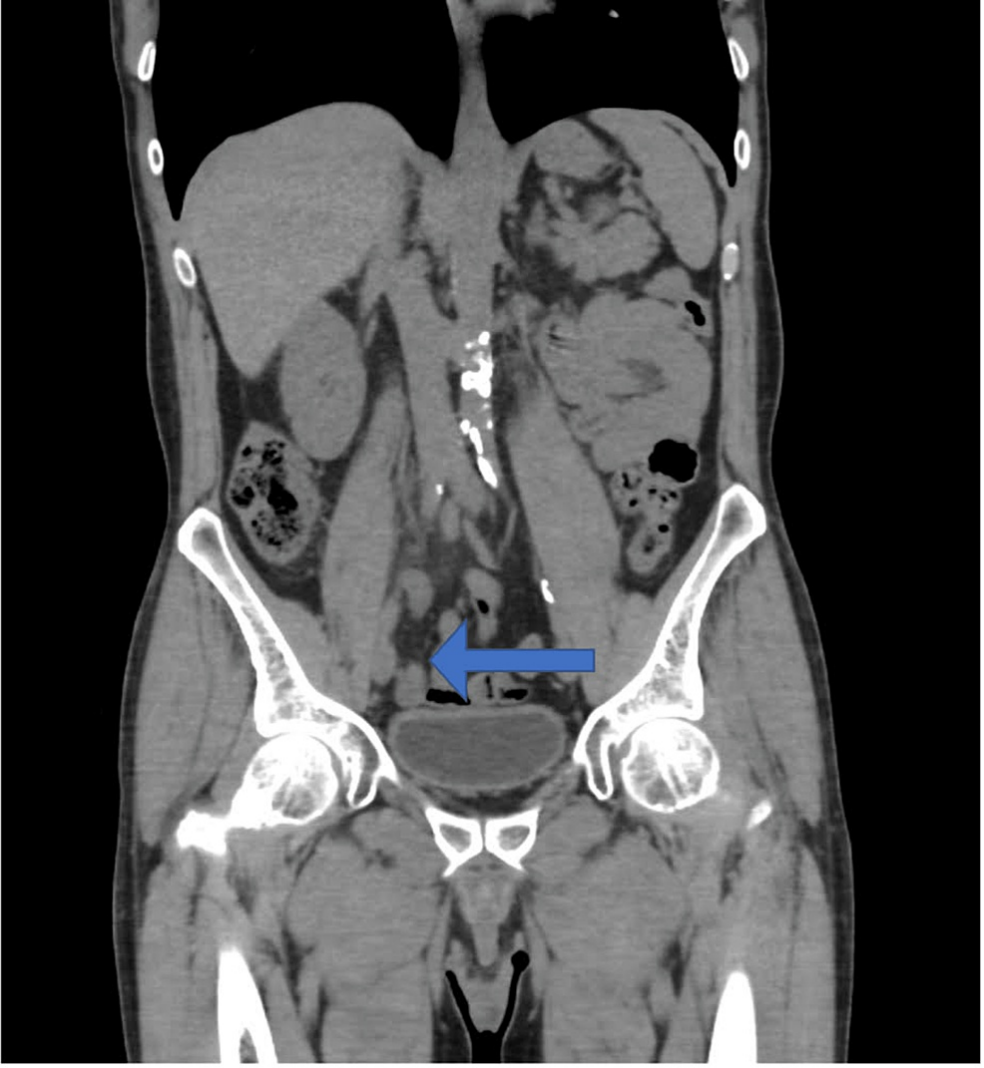
Coronal computed tomography scan done on April 24th.

**Figure 2:**
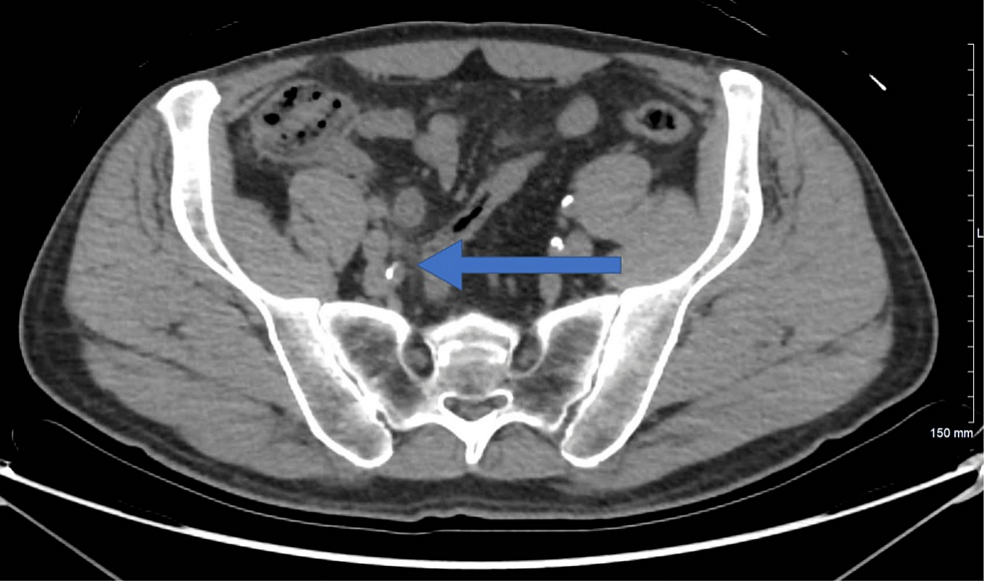
Axial computed tomography scan done on April 24th.

**Figure 3:**
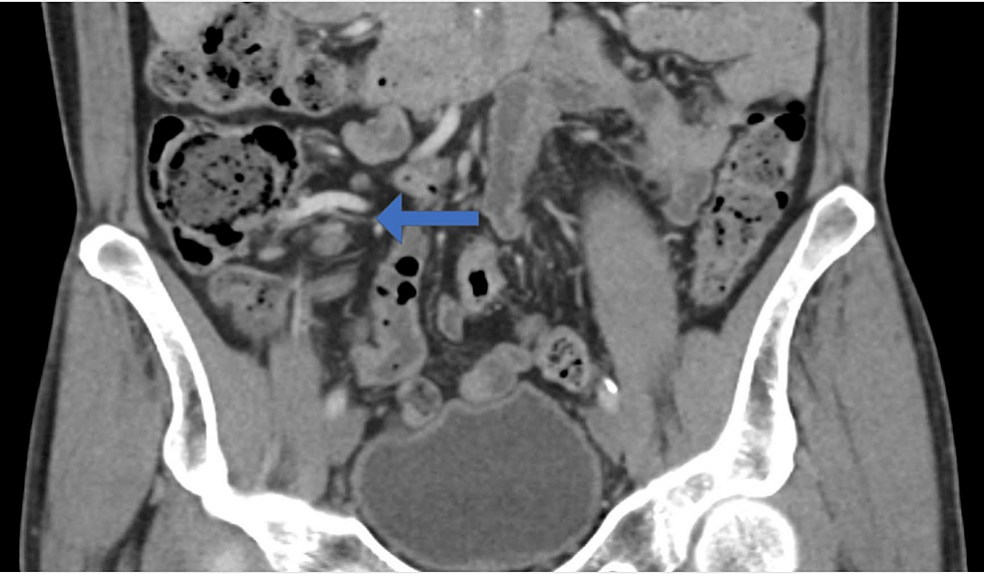
Coronal computed tomography scan done on June 12th.

**Figure 4:**
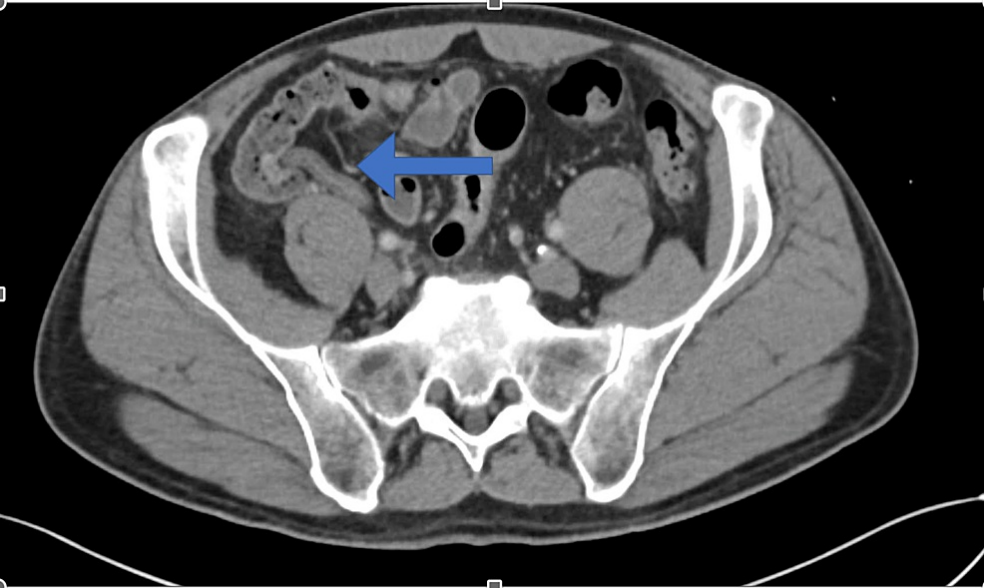
Axial computed tomography scan done on June 12th.

A 61-year-old male patient, a known case of renal calculi following up with the nephrology department, presented to the clinic on April 24 for a non-obstructing renal calculus that was diagnosed previously. The patient underwent a computed tomography (CT) scan of the abdomen and pelvis which showed some incidental appendiceal findings. The appendix was found to be diffusely thickened and surrounded by mild fat stranding (Figures 1, 2). According to the CT scan report, this finding was related to early or resolving appendicitis. Based on the patient’s status of being free of symptoms of abdominal pain that he had experienced a few days back and normal white blood cells, his surgeon advised that it was resolving appendicitis. The patient was discharged home and advised to seek medical attention right away if he experienced similar pain. On May 9, the patient presented to the clinic with a two-day history of continuous periumbilical pain without nausea or vomiting. During this presentation, the patient was offered non-steroidal anti-inflammatory drugs (NSAIDs), following which the pain subsided and his symptoms resolved. The patient was advised to undergo a CT scan to re-evaluate his appendix. On June 12, the patient underwent a CT scan of the abdomen and pelvis to evaluate his appendix. The bowel loops were grossly unremarkable, and the appendix was of normal thickness, with the resolution of previously seen minimal surrounding inflammation (Figures 3, 4). Ten days later, the patient presented to the emergency department complaining of continuous severe periumbilical pain that had started one day ago and was similar to his previous recurrent abdominal pain. The pain did not radiate to the RLQ and was not associated with any fever, chills, nausea, vomiting, fatigue, or anorexia. On examination, there was no abdominal distention, and the patient was free of RLQ tenderness, guarding, and rebound tenderness. The patient’s vital signs and investigations were not significant, except for high inflammatory markers (Table 1).

**Table 1:** Vital signs and investigations on June 22nd.

Test	Result	Normal range
Temperature (°C)	36.4	36.1–37.2
Respiratory rate (breaths/minute)	28	12–20
Heart rate (beats/minute)	74	60–100
Blood pressure (mmHg)	112/67	>120/80
Oxygen saturation	99% on room air	95–100%
Weight (kg)	80.9	NA
Height (cm)	178.5	NA
Body mass index (kg/m^2^)	25.39	18.5–24.9
C-reactive protein (mg/L)	90.1	0–5
Total bilirubin (µmol/L)	11.8	3.4–22.1
Hemoglobin (g/dL)	15.6	13–18
White blood cells (×10^9^/L)	8.3	4.0–11.0
Platelets (×10^9^/L)	146	150–450

**Figure 5:**
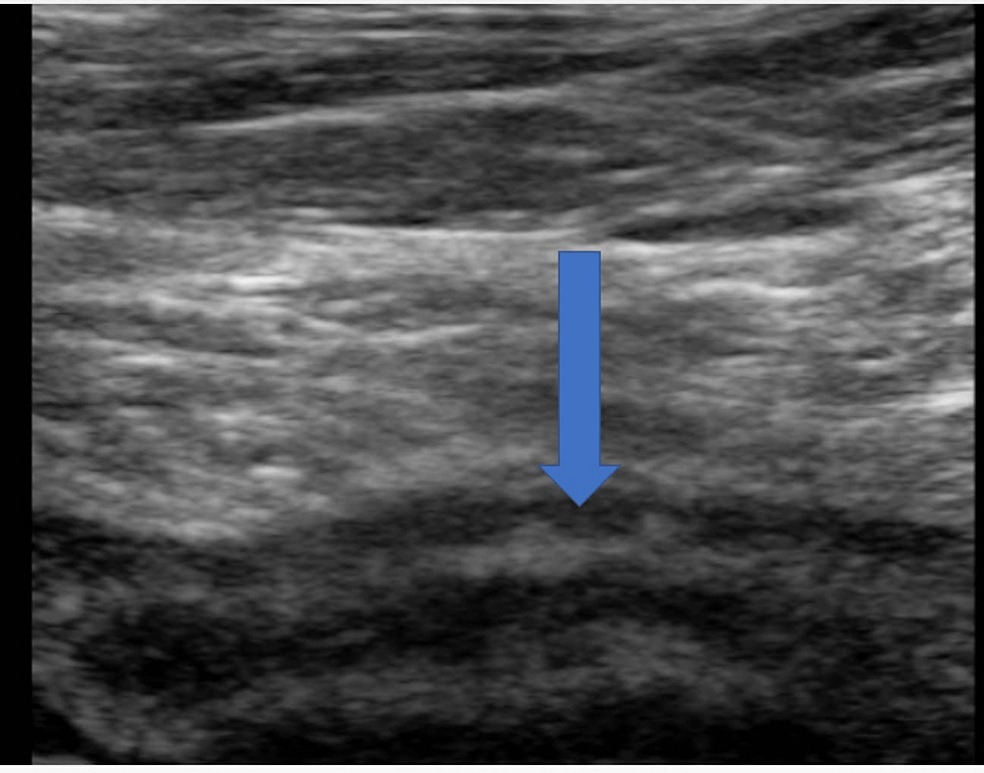
Longitudinal image of the right inguinal area ultrasound done on June 22nd.

**Figure 6:**
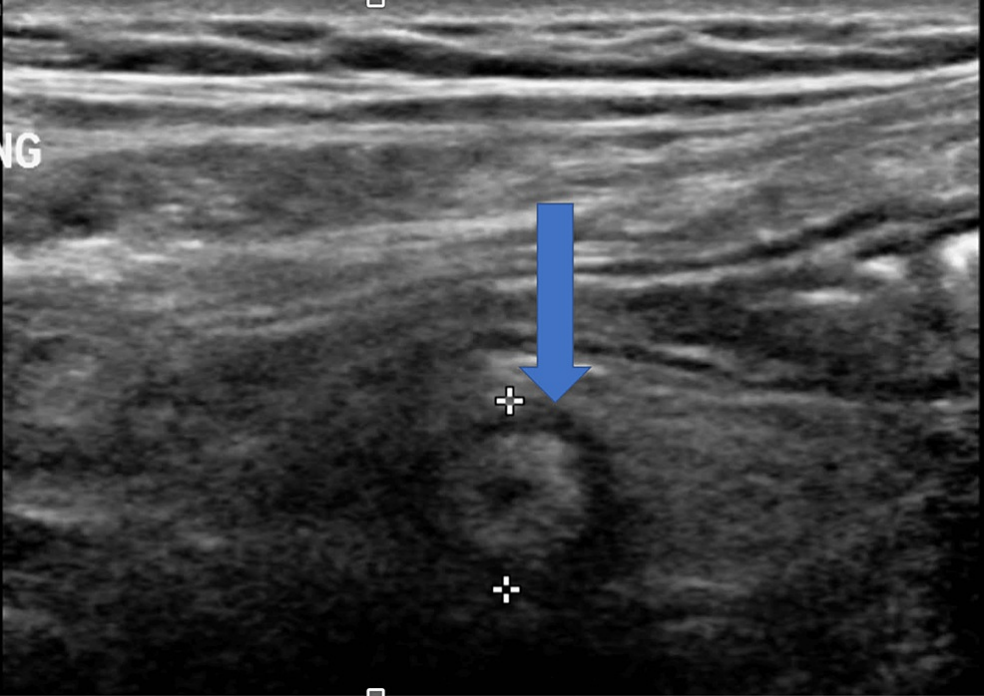
Transverse image of the right inguinal area ultrasound done on June 22nd.

The patient underwent an ultrasound (US) at this encounter, and the appendix was found to be enlarged (Figures 5, 6). Given that the appendix was normal on a recent CT scan, this acute presentation with radiological evidence of enlargement was suggestive of mild acute appendicitis. The patient underwent laparoscopic exploration for possible appendectomy under general anesthesia. An infraumbilical incision was made and an entry into the peritoneum was confirmed visually. No bowel was noted on the vanity of the incision. The abdomen was insufflated, and the patient tolerated the insufflation well. The appendix was noted to be inflamed and resected. The patient tolerated the operation well and was taken to the post-anesthesia and recovery room in stable condition. The appendix was sent for histopathology, and a final diagnosis was made by the general surgery department as acute suppurative appendicitis with peri-appendicitis.

The pathology report mentioned that the dimensions of the appendix were 6 × 1.3 cm with congested serosa. There was no perforation or fecalith. The lumen was 0.4 cm dilated and obliterated. The mesoappendix fat dimensions were 4 × 2 × 0.3 cm.

## Discussion

The pathogenesis of acute appendicitis is widely understood; however, the presence of chronic appendicitis appears to be debatable among many specialists [8]. Chronic appendicitis is a diagnosis that many practitioners are unfamiliar with, and there are no definitive diagnostic criteria. The pathophysiology of chronic appendicitis is vague. In acute appendicitis, the obstruction of the appendiceal lumen due to bacterial overgrowth results in the distension of the appendix causing inflammation, ischemia, and perforation [9]. However, some studies speculate that chronic appendicitis may be caused by either partial obstruction of the appendiceal lumen or excessive mucus production in the appendix [4,9]. The diagnosis is often made after appendectomy and is based on the histological findings of chronic inflammatory changes [4]. The usual manifestation of acute appendicitis is 48 hours of periumbilical pain that radiates to the right iliac fossa. It is frequently accompanied by nausea, vomiting, anorexia, abdominal guarding, rebound tenderness, and neutrophil-predominant leukocytosis [2,10]. Recurring abdominal pain for more than seven days has previously been proposed to differentiate acute from chronic appendicitis [11]. However, chronic appendicitis often manifests as less severe, constant abdominal discomfort that lasts longer than the typical duration of acute appendicitis. It may last weeks, months, or even years [10]. This can result in misdiagnosis and diagnostic delays due to the milder sequel of chronic appendicitis symptoms.

In some cases, patients were prescribed antibiotics for abdominal pain before performing appropriate imaging studies, assuming other causes, such as urinary tract infection, pelvic inflammatory diseases, or other differentials, as the final diagnosis. Although this intervention relieves their pain, it delays the accurate diagnosis of chronic appendicitis [2,6,12,13]. CT imaging has been proposed in the literature as the main imaging tool for diagnosing chronic appendicitis [14].

In our case, histopathological results showed appendiceal dimensions of 6 × 1.3 cm, which were similar to those reported in previous cases of chronic appendicitis [5,13]. Contrary to acute appendicitis, chronic appendicitis is not considered a surgical emergency [2]. However, the diagnosis may be overlooked or delayed due to unusual presentation or prior antibiotic therapy, which may result in infection remission. Misdiagnosis can result in significant consequences such as perforation, abscess development, and peritonitis [2]. It is critical to rule out chronic appendicitis by a CT scan if atypical symptoms, such as in our case, are present.

## Conclusions

We presented the case of a 61-year-old male with incidental appendiceal CT scan findings showing signs of appendicitis. History demonstrated that he had intermittent abdominal pain for a long time. He was managed supportively with NSAIDs which alleviated his symptoms. Afterward, he came back to the clinic complaining of abdominal pain, and his CT scan findings were inconclusive. The patient then presented a few days later to the emergency room complaining of continuous periumbilical pain with no other associations. US imaging showed diffuse thickening of the appendix. The patient underwent laparoscopic exploration and the appendix was resected because of its inflammation. After histopathology assessment, a final diagnosis was made by the general surgery department as acute suppurative appendicitis with peri-appendicitis.
